# Intra-articular interleukin-1 receptor antagonist (IL1-ra) microspheres for posttraumatic osteoarthritis: in vitro biological activity and in vivo disease modifying effect

**DOI:** 10.1186/s40634-016-0054-4

**Published:** 2016-08-19

**Authors:** Khaled A. Elsaid, Anand Ubhe, Ziyad Shaman, Gerard D’Souza

**Affiliations:** 1 Department of Pharmaceutical Sciences, School of Pharmacy-Boston, MCPHS University, Boston, MA USA; 2 Biomedical and Pharmaceutical Sciences, Chapman University School of Pharmacy, 9401 Jeronimo Road, Irvine, CA 92618 USA

**Keywords:** Interleukin-1 receptor antagonist, Posttraumatic osteoarthritis, IL1 ra microspheres, Bioactivity of IL1ra

## Abstract

**Background:**

Interleukin-1 receptor antagonist (IL-1 ra) can be disease-modifying in posttraumatic osteoarthritis (PTOA). One limitation is its short joint residence time. We hypothesized that IL-1 ra encapsulation in poly (lactide-co-glycolide) (PLGA) microspheres reduces IL-1 ra systemic absorption and provides an enhanced anti-PTOA effect.

**Methods:**

*IL-1 ra release kinetics and biological activity:* IL-1 ra encapsulation into PLGA microsphere was performed using double emulsion solvent extraction. Lyophilized PLGA IL-1 ra microspheres were resuspended in PBS and supernatant IL-1 ra concentrations were assayed. The biological activity of IL-1 ra from PLGA IL-1 ra microspheres was performed using IL-1 induced lymphocyte proliferation and bovine articular cartilage degradation assays. *Systemic absorption of IL-1 ra following intra-articular (IA) injection of PLGA IL-1 ra or IL-1 ra:* At 1, 3, 6, 12 and 24 h following injection of 50 μl PLGA IL-1 ra (*n* = 6) or IL-1 ra (*n* = 6), serum samples were collected and IL-1 ra concentrations were determined. *Anterior cruciate ligamenttransection (ACLT) and IA dosing:* ACLT was performed in 8–10 week old male Lewis rats (*n* = 42). PBS (50 μl; *n* = 9), IL-1 ra (50 μl; 5 mg/ml; *n* = 13), PLGA IL-1 ra (50 μl; equivalent to 5 mg/ml IL-1 ra; *n* = 14) or PLGA particles (50 μl; *n* = 6) treatments were performed on days 7, 14, 21 and 28 following ACLT. *Cartilage and synovial histopathology:* On day 35, animal ACLT joints were harvested and tibial cartilage and synovial histopathology scoring was performed.

**Results:**

Percent IL-1 ra content in the supernatant at 6 h was 13.44 ± 9.27 % compared to 34.16 ± 12.04 %, 47.89 ± 12.71 %, 57.14 ± 11.71 %, and 93.90 ± 8.50 % at 12, 24, 48 and 72 h, respectively. PLGA IL-1 ra inhibited lymphocyte proliferation and cartilage degradation similar to IL-1 ra. Serum IL-1 ra levels were significantly lower at 1, 3, and 6 h following PLGA IL-1 ra injection compared to IL-1 ra. Cartilage and synovial histopathology scores were significantly lower in the PLGA IL-1 ra group compared to PBS and PLGA groups (*p* < 0.001).

**Conclusions:**

IL-1 ra encapsulation in PLGA microspheres is feasible with no alteration to IL-1 ra biological activity. PLGA IL-1 ra exhibited an enhanced disease-modifying effect in a PTOA model compared to similarly dosed IL-1 ra.

## Background

Posttraumatic osteoarthritis (PTOA) is estimated to affect approximately 5.6 million individuals in the US with anterior cruciate ligament (ACL) ruptures accounting for a considerable proportion of PTOA disease burden. Factors e.g. direct cartilage injury; inflammation and joint instability converge to trigger early cartilage degeneration. Proinflammatory cytokines e.g. interleukin-1 (IL1), IL6, and tumor necrosis factor alpha (TNFα) are elevated in synovial fluid aspirates from patients following ACL injury and play an important role in the initial disease process (Cameron et al. 1994; Catterall et al. 2010; Elsaid et al. 2008; Higuchi et al. 2006; Irie et al. 2003). Interleukin-1 receptor antagonist (IL-1 ra) is a promising treatment that has shown a disease-modifying activity in pre-clinical animal PTOA models (Allen et al. 2011; Caron et al. 1996). Clinically, biologic IL-1 inhibitor therapies have failed to show a sustained clinical effect contributed by a short joint residence time following intra-articular administration (Chevalier et al. 2009; Cohen et al. 2011).

The joint residence time of small molecules and large polymers following intra-articular administration is in the order of hours (Owen et al. 1994; Vugmeyster et al. 2012). Encapsulation of therapeutic agents in pharmaceutical carriers increases joint residence time and enhances the pharmacologic activity of these agents (Pradal et al. 2015; Vanniasinghe et al. 2014; Evans et al. 2014). Poly (lactide-co-glycolide) (PLGA) is an inert biodegradable polymer that has been successfully used to encapsulate small molecules, nucleic acids and large recombinant proteins (Gaignaux et al. 2012; Jilek et al. 2005; Wang et al. 2012; Wischke & Schwendeman 2008; Nie et al. 2015). Specifically, PLGA encapsulation was used to provide sustained release of non-steroidal anti-inflammatory drugs, chondroitin sulfate and anti-TNFα therapies in pre-clinical arthritis models (Higaki et al. 2005; Jiang et al. 2011; Présumey et al. 2012).

In this study, we explored the feasibility of encapsulating IL-1 ra in PLGA and we studied the biological activity of IL-1 ra released from PLGA IL-1 ra microspheres. Furthermore, we studied the disease-modifying effect of PLGA IL-1 ra microspheres in a PTOA rat model of ACL transection.

## Methods

### Preparation and characterization of IL-1 ra microspheres

Anakinra (Kineret), a 100 mg per 0.67 ml pre-filled syringe for subcutaneous administration, is the form of IL-1 ra used in this study. IL-1 ra microspheres were prepared by a double emulsion solvent extraction method as previously described (Butoescu et al. 2008). IL-1 ra (5 mg; 33 μl) and 3 mg/100 μl of iron oxide dispersion were mixed together and bath sonicated for 30 s to get a uniform dispersion. This aqueous phase was emulsified with 1 ml of 100 mg/ml solution of PLGA: lactide: glycolide 85:15, MW 50,000–75,000 (Sigma Aldrich, USA) in ethyl acetate. The resultant emulsion was then dispersed in 2 ml of 2 % polyvinyl alcohol (PVA) solution in distilled water by probe sonication for 15 s. This double emulsion was diluted with 10 ml of 0.3 % PVA solution in distilled water and stirred mechanically for 4 h to yield the particles. The resultant particles were magnetically separated and washed with distilled water and the lyophilized. Lyophilized particles were stored at 4 °C and were reconstituted as needed in PBS. Particle size distribution and surface charge were analyzed using 90 Plus particle size analyzer (Brookhaven Instrument Corporation, USA). Representative preparations were imaged using a scanning electron microscope. The incorporation efficiency of IL-1 ra in PLGA microparticles was determined using lyophilized PLGA IL-1 ra from 4 different preparations. A total of 1 mg of PLGA IL-1 ra microparticles was reconstituted in 1 ml of 5 % SDS in 0.1 N NaOH and incubated for 15 h at room temperature, where by all powder dissolved. Total protein content was determined using the BCA colorimetric assay (ThermoFisher Scientific, USA) and empty PLGA particles were processed in a similar manner and used as a blank. IL-1 ra incorporation was expressed as a ratio of the total measured protein in microparticles to the total protein used in the formulation process. The data represents the average IL-1 ra percent incorporation from the 4 preparations. The release of IL-1 ra from the PLGA micrsopheres was evaluated using the equivalent of 5 mg of PLGA IL-1 ra from 4 different preparations following reconstitution in 1 ml of PBS and incubation at room temperature with shaking. At 6, 12, 24, 48 and 72 h following reconstitution, particles were centrifuged at 10,000 rpm and the supernatant was sampled. The IL-1 ra concentration in the supernatant was quantified using a commercially available ELISA (R&D Systems, USA) and was expressed as the ratio of IL-1 ra content in the supernatant to the total expected amount of incorporated IL-1 ra. Data represents the average IL-1 ra percent release from the 4 preparations.

### Biological activity of IL-1 ra released from PLGA IL-1 ra microspheres using human lymphocyte proliferation assay

We have utilized RPMI 1788 human lymphocytes (American Type Cell Culture, USA) to evaluate the dose-dependent proliferation-inhibitory effect of IL-1 ra released from PLGA IL-1 ra microspheres (Shamji et al. 2007; Vandenabeele et al. 1990). RPMI 1788 cells were grown in RPMI 1640 culture medium (Sigma Aldrich, USA) supplemented with 10 % fetal bovine serum, 2 mM L-glutamine, 0.05 mM β mercaptoethanol, 1 mM sodium pyruvate, 10 mM HEPES and 1 % penicillin/streptomycin. Using sterile 96-well plates (Corning, USA), a total of 1,000 cells in 80 μl media was added to each well followed by the addition of recombinant human IL1β (R&D Systems) at a final concentration of 500 pg/ml with or without the addition of iron-oxide containing PLGA, IL-1 ra or PLGA IL-1 ra (pooled from 4 different preparations) at a final concentration of 1 ng/ml or 25 ng/ml. The final volume in each well was 100 μl. RPMI cells that received no IL1β, PLGA IL-1 ra or IL-1 ra served as controls. Plates were incubated at 37 °C in 5 % CO_2_ for 72 h followed by assaying cell proliferation using Cell Titer 96 non-radioactive cell proliferation kit (Promega, USA). The percent inhibition of cell proliferation was calculated for each concentration of PLGA IL-1 ra or IL-1 ra and values were compared across treatments. Data represents the average ± SD of three independent experiments, each with duplicate wells per group.

### Biological activity of IL-1 ra released from PLGA IL-1 ra microspheres using IL-1α stimulated bovine articular cartilage explant model

Full-thickness bovine articular cartilage explants (12 mm in diameter) were harvested from the femoral condyles of skeletally mature steers with no signs of gross cartilage degeneration. Cartilage explants were maintained in serum-free Dulbecco’s Modified Eagle Medium (DMEM; Sigma Aldrich) supplemented with 1 % penicillin/streptomycin. Cartilage explants were stimulated with 10 ng/ml of recombinant human IL-1α (R&D Systems) with or without iron-oxide containing PLGA, PLGA IL-1 ra at 20 ng/ml and 100 ng/ml (pooled from 4 different preparations) or IL-1 ra at 20 ng/ml and 100 ng/ml (*n* = 6 in each group). Media collections and supplementation with IL-1α and the drug were performed at 3, 5 and 7 days. Cumulative sulfated glycosaminoglycan (sGAG) released over 7 days was measured using dimethylmethylene blue (DMMB) binding assay and compared across groups (Mort & Roughley 2007).

### Serum IL-1 ra levels following intra-articular administration of PLGA IL-1 ra or IL-1 ra

A total of 50 μl of IL-1 ra (5 mg/ml) or 50 μl of PLGA IL-1 ra (equivalent to 5 mg/ml of IL-1 ra) were administered intra-articularly to naive male Lewis rats (8–10 weeks; *n* = 6 in each group) with an implanted jugular vein catheter under isoflurane anesthesia. At 1, 3, 6, 12 and 24 h, blood was sampled via the jugular vein catheter and serum IL-1 ra concentrations were determined using a commercially-available ELISA (Life Technologies, USA).

### ACLT in the rat

ACLT was performed in 8–10 weeks old male Lewis rats (*n* = 42). Following anesthesia with intraperitoneal Ketamine and Dexmedetomidine, the right knee joint skin was shaved, disinfected with a topical antiseptic, and a lateral skin incision was made to access the joint capsule. A lateral incision was performed along the patellar tendon to open the capsule and expose the ACL. Transecting the ACL was performed using a # 11 surgical blade and a positive anterior draw confirmed ACLT. Closure of the capsule and skin was performed using biodegradable sutures. All surgeries were performed by author KE and all required approvals were obtained from MCPHS University IACUC committee prior to the commencement of the study. We have also utilized 4 control age-matched male Lewis rats.

### Drug treatments

Following ACLT, animals were randomly assigned to the following groups: PBS (*n* = 9), IL-1 ra (*n* = 13), PLGA IL-1 ra (*n* = 14) and PLGA (*n* = 6). Animals received intra-articular injections of PBS (50 μl), IL-1 ra (5 mg/ml; 50 μl), PLGA IL-1 ra (equivalent to 5 mg/ml of IL-1 ra; 50 μl; pooled from 4 different preparations) or empty iron oxide-containing PLGA particles (100 mg/ml; 50 μl) on days 7, 14, 21, and 28 following ACLT. Animals were anesthetized with isoflurane and injections were performed through the patellar tendon of the operated knee joint.

### Histological analyses and scoring

Animals were euthanized on day 35 following ACLT and their ACLT joints were harvested. Following decalcification, paraffin-embedded coronal sections were taken through the weight-bearing areas of the ACLT joints of each animal. Microtomed sections were collected every 250 μm and only the sections showing the tibial plateaus, femoral condyles and the menisci were considered. A total of 4 histological sections were stained with Safranin O/Fast Green for assessment of cartilage integrity and 4 adjacent histological sections were stained with Hematoxyln and Eosin (H&E) for synovitis scoring. Cartilage histopathology scoring was performed using a previously described quantitative assessment tool (Pritzker et al. 2006). The overall score is a product of the cartilage degeneration grade and stage. Cartilage degeneration grade ranged from 0 indicating intact cartilage surface and morphology, normal architecture and proteoglycan staining and appropriate chondrocyte orientation to 5 indicating cartilage denudation, full-thickness cartilage loss and formation of repair tissue and underlying bone changes. The cartilage degeneration stage ranged from stage 1 involving <10 % of cartilage surface/area to stage 4 involving > 50 % of cartilage surface/area. Scoring was performed by two blinded investigators and a consensus score was reported. Investigators scored the medial and lateral sides of the 4 histological sections and the overall joint score was the mean of the 8 scores. Synovial histopathology scoring was performed using the criteria reported previously (Cake et al. 2008) and included examination of intimal hyperplasia, inflammatory cell infiltration, subintimal fibrosis and vascularity with scores ranging from 0 to 3 for each criterion and a range of aggregate scores between 0 and 12.

### Determination of urinary CTXII (uCTXII) levels in urines of ACLT animals from different groups

On day 34 following ACLT, animals were housed in metabolic cages and 24 h urine collection was performed. Urine samples were centrifuged at 10,000 rpm and stored at −20 °C until analysis. The uCTXII levels were quantified using the Urine Pre-Clinical Cartilaps ELISA (Immunodiagnostic Systems, USA). Urinary creatinine was measured colorimetrically using a commercially available assay kit (Sigma Aldrich). The uCTXII levels were normalized to urinary creatinine and expressed as μg per nano moles of creatinine.

### Statistical analyses

Percent IL-1 ra released from PLGA microspheres, percent inhibition of lymphocyte proliferation, sGAG release, and IL-1 ra serum concentrations were plotted as mean ± standard deviation (SD). Cartilage histopathology scores, synovial pathology scores and uCTXII levels were reported using box plots. Multiple group comparisons for variables that satisfy equal variance and normality assumptions were performed by one-way analysis of variance (ANOVA) with Tukey’s post-hoc pairwise comparisons. Variables that did not satisfy the two assumptions were compared using ANOVA on the ranks. Serum IL-1 ra concentrations following IL-1 ra or PLGA IL-1 ra administrations were compared using Student’s *t*-test. Statistical significance was set a priori at α = 0.05.

## Results

### PLGA IL-1 ra microsphere characterization and IL-1 ra release

**Fig. 1:**
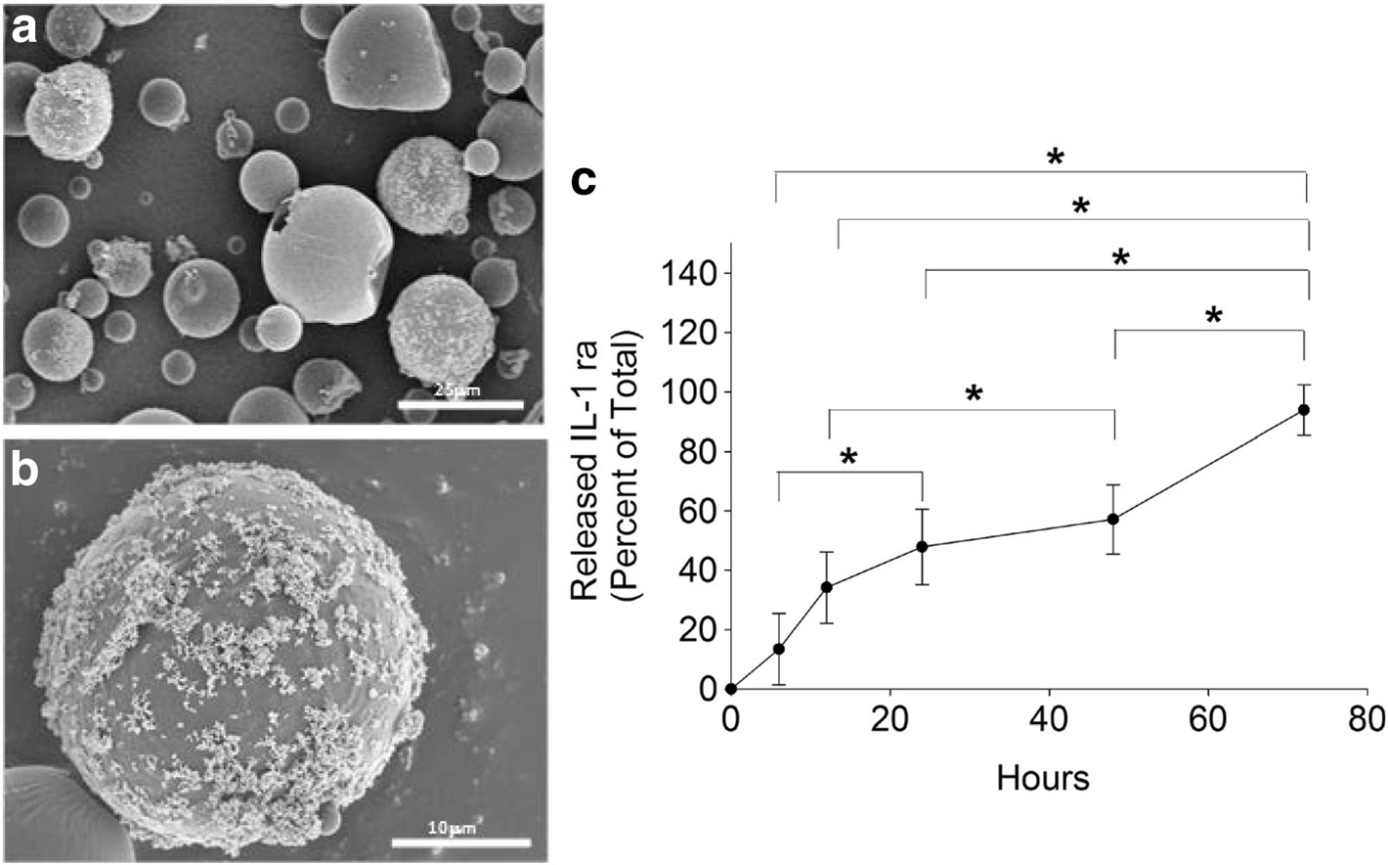
IL-1 ra containing PLGA microspheres. **a**, **b** Representative scanning electron micrographs of PLGA IL-1 ra microspheres at different magnifications. **c** Release of IL-1ra from PLGA-IL1 ra microspheres over time. Cumulative IL-1 ra release at 72 h was significantly higher than cumulative release at 6, 12, 24 and 48 h. Similarly, cumulative IL-1 ra release at 24 and 48 h was significantly higher than cumulative release at 6 and 12 h, respectively. *p < 0.001. Data represents the average ± SD of 4 different preparations

Representative scanning electron micrographs of PLGA IL-1 ra is shown in Fig. 1a and b. Presence of iron oxide is observed in the prepared particles. Additionally, particles demonstrated some degree of size heterogeneity. The mean effective diameter for PLGA particles was 1.9 ± 0.5 μm, while the mean effective diameter of PLGA IL-1 ra particles was 5.1 ± 2.1 μm. The particle zeta potential of PLGA IL-1 ra was found to be 5.44 ± 0.77 mV. The average percent incorporation of IL-1 ra into iron-oxide containing PLGA particles was 16.44 ± 3.96 %. The time-dependent release of IL-1 ra from PLGA IL-1 ra is shown in Fig. 1c. The percent of IL-1 ra content in the supernatant at 6 h following reconstitution was 13.44 ± 9.27 % compared to 34.16 ± 12.04 %, 47.89 ± 12.71 %, 57.14 ± 11.71 %, and 93.90 ± 8.50 % at 12, 24, 48 and 72 h, respectively. Cumulative IL-1 ra release at 72 h was significantly higher than cumulative release at 6, 12, 24 and 48 h (*p* < 0.001). Cumulative IL-1 ra release at 48 h was significantly higher than cumulative release at 12 h (*p* < 0.001). Similarly, cumulative IL-1 ra release at 24 h was significantly higher than cumulative release at 6 h (*p* < 0.001).

### Biological activity of IL-1 ra from PLGA IL-1 ra

**Fig. 2:**
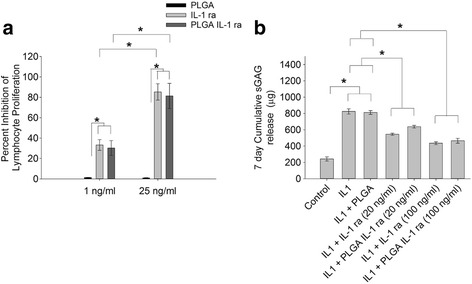
Impact of PLGA incorporation on IL-1 ra biological activity. **a** Inhibition of interleukin-1 beta (IL-1β)-induced human lymphocyte proliferation by IL-1 ra or PLGA-encapsulated IL-1 ra (PLGA IL-1 ra). The percent inhibition of lymphocyte proliferation following treatment with 25 ng/ml of IL-1 ra or PLGA IL-1 ra was significantly higher than the percent inhibition of lymphocyte proliferation following treatment with 1 ng/ml of IL-1 ra or PLGA IL-1 ra. There was no significant difference in percent inhibition of lymphocyte proliferation between IL-1 ra and PLGA IL-1 ra treatments at 1 ng/ml or 25 ng/ml. *p < 0.001. Data represents the average ± SD of three independent experiments, each with duplicate wells per group. PLGA IL-1 ra was pooled from 4 different preparations**. b** cumulative sulfated glycosaminoglycan (sGAG) release, over 7 days, from control bovine articular cartilage explants (control), interleukin-1 α-stimulated (IL-1α), IL-1α stimulated + treatment with IL-1 ra at 20 ng/ml (IL-1 + IL-1 ra (20 ng/ml)), IL-1α stimulated + treatment with PLGA IL-1 ra at 20 ng/ml (IL-1 + PLGA IL-1 ra (20 ng/ml)), IL-1α stimulated + treatment with IL-1 ra at 100 ng/ml (IL-1 + IL-1 ra (100 ng/ml)) and IL-1α stimulated + treatment with PLGA IL-1 ra at 100 ng/ml (IL-1 + PLGA IL-1 ra (100 ng/ml)). (*n* = 6 in each group). IL-1α treatment resulted in a significantly higher sGAG cartilage release compared to control. Treatment with IL-1 ra or PLGA IL-1 ra at 20 ng/ml resulted in a significant reduction in sGAG release compared to IL-1α alone. Similarly, treatment with IL-1 ra or PLGA Il-1 ra at 100 ng/ml resulted in a significant reduction in sGAG release compared to IL-1 ra alone. There was no significant difference in sGAG release between IL-1 ra and PLGA IL-1 ra at 20 ng/ml or 100 ng/ml. *p < 0.001. Data represents the average ± SD of 6 explants per group. PLGA IL-1 ra was pooled from 4 different preparations

The impact of PLGA encapsulation on inhibition of human lymphocyte proliferation by IL-1 ra is shown in Fig. 2a. At the 1 ng/ml and 25 ng/ml concentrations, no significant differences were found between PLGA IL-1 ra and IL-1 ra treatments. The percent inhibition of lymphocyte proliferation following treatment with 25 ng/ml of PLGA IL-1 ra or IL-1 ra was significantly higher than the percent inhibition of lymphocyte proliferation following treatment with 1 ng/ml of PLGA IL-1 ra or IL-1 ra (*p* < 0.001). Treatment with PLGA particles alone did not inhibit lymphocyte proliferation.

The impact of PLGA encapsulation on inhibition of sGAG loss from bovine articular cartilage explants by IL-1 ra is shown in Fig. 2b. IL-1α treatment resulted in a significant increase in the cumulative release of sGAG compared to untreated control (*p < 0.001*). Treatment with PLGA particles alone did not alter sGAG release from bovine explants. Treatment with PLGA IL-1 ra or IL-1 ra at 20 ng/ml concentration resulted in a significant reduction in sGAG release compared to IL-1α treatment (*p < 0.001*). Additionally, 20 ng/ml IL-1 ra treatment resulted in a significant reduction in sGAG release compared to 20 ng/ml PLGA IL-1 ra treatment (*p = 0.021*). At 100 ng/ml, PLGA IL-1 ra and IL-1 ra treatments resulted in a significant reduction in sGAG release compared to IL-1α treatment (*p < 0.001*) with no difference between cumulative sGAG release between PLGA IL-1 ra and IL-1 ra treatments.

### IL-1 ra serum levels following intra-articular administration of PLGA IL-1 ra or IL-1 ra

**Fig. 3:**
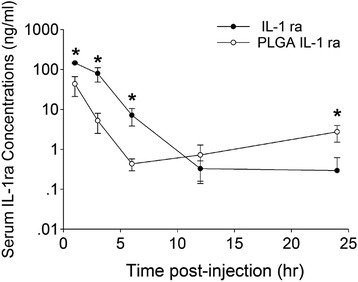
Serum IL-1 ra concentrations following intra-articular administration of IL-1 ra or PLGA IL-1 ra. *p < 0.001. Data represents the average ± SD of 6 animals in each experimental group

Serum IL-1 ra concentrations following intra-articular administration of PLGA IL-1 ra or IL-1 ra is shown in Fig. 3. At 1, 3 and 6 h, serum IL-1 ra levels were significantly higher in the IL-1 ra group than the corresponding levels in the PLGA IL-1 ra group (*p < 0.001*). At 12 h, there was no significant difference in serum IL-1 ra between PLGA IL-1 ra and IL-1 ra treatments. At 24 h, serum IL-1 ra levels in the PLGA IL-1 ra group was significantly higher (*p < 0.001*) than the levels in the IL-1 ra group.

### Disease-modifying effect of PLGA IL-1 ra in rat ACLT model

**Fig. 4:**
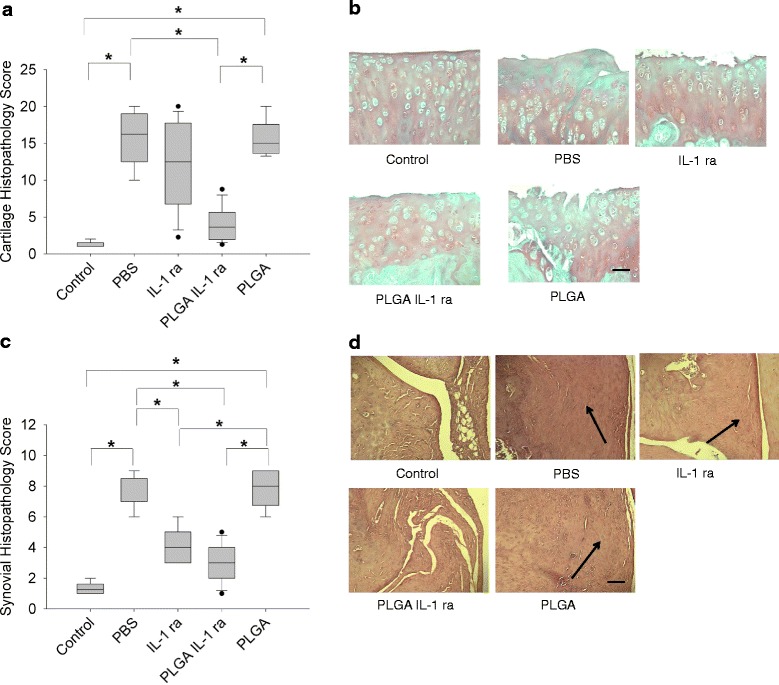
The disease-modifying activity of IL-1 ra or PLGA IL-1 ra in the rat ACLT model. **a** Cartilage histopathology scores of control joints and joints undergoing ACLT and receiving weekly intra-articular injections of PBS (*n* = 9), IL-1 ra (*n* = 13), PLGA-encapsulated IL-1 ra (PLGA IL-1 ra; *n* = 14) or PLGA microspheres (PLGA; *n* = 6) for 4 weeks starting one week following ACLT. PBS treated ACLT joints exhibited a significantly higher mean cartilage histopathology score compared to control. Similarly, PLGA-treated ACLT joints exhibited a significantly higher mean cartilage histopathology score compared to control. PLGA IL-1 ra treated ACLT joints demonstrated a significantly lower mean cartilage histopathology score compared to PBS or PLGA-treated ACLT joints. *p < 0.001. Data represents average ± SD. **b** Representative Safranin O/Fast Green stained tibial plateau cartilage specimens from control, ACLT animals receiving PBS, IL-1 ra, PLGA-IL1 ra or PLGA. ACLT joints exhibited loss of Safranin O staining indicative of glycosaminoglycan loss, clefts extending into the middle zone and hypocellularity. PLGA IL-1ra treatment reduced glycosaminoglycan loss and the number and depth of cartilage tissue fissures. Scale = 100 μm. **c** Synovial histopathology scores of control joints and joints undergoing ACLT and receiving weekly intra-articular injections of PBS (*n* = 9), IL-1 ra (*n* = 13), PLGA IL-1 ra (*n* = 14) or PLGA (*n* = 6) for 4 weeks starting one week following ACLT. PBS treated ACLT joints exhibited a significantly higher mean synovial histopathology score compared to control. Similarly, PLGA-treated ACLT joints exhibited a significantly higher mean synovial histopathology score compared to control. IL-1 ra treated ACLT joints exhibited a significantly lower mean synovial histopathology score compared to PBS or PLGA-treated ACLT joints. Similarly, PLGA Il-1 ra treated ACLT joints exhibited a significantly lower mean synovial histopathology score compared to PBS or PLGA-treated ACLT joints. *p < 0.001. Data represents average ± SD. **d** Representative H&E-stained synovia specimens from control, ACLT animals receiving PBS, IL-1 ra, PLGA IL-1 ra or PLGA. Arrows point to synovial hyperplasia evident in ACLT animals treated with PBS, IL-1 ra and PLGA

Cartilage histopathology scores of Safranin O-stained specimens from the different experimental groups are presented in Fig. 4a. PBS-treated ACLT cartilage exhibited a significantly higher degree of cartilage degeneration compared to non-surgical controls (*p* < 0.001). PLGA-treated ACLT cartilage exhibited a comparable degree of cartilage degeneration compared to PBS-treated ACLT cartilage. IL-1 ra treatment did not significantly reduce cartilage degeneration compared to PBS treatment. On the contrary, PLGA IL-1 ra treatment significantly reduced cartilage degeneration compared with PBS or PLGA treatments (*p* < 0.001). There was no significant difference in cartilage histopathology scores between control and PLGA IL-1 ra groups.

Representative Safranin O and H&E stained histological sections are presented in Fig. 4b. Consistently, PLGA IL-1 ra treatment resulted in cartilage tissue architectural integrity, with some occasional fissures and surface erosion that did not extend into the middle zone as well as sGAG staining. On the contrary, PBS and PLGA treatments displayed cartilage surface loss, loss of sGAG staining and clefts, extending into the middle zone. In animals treated with IL-1 ra, a significant variation in the extent of cartilage tissue degeneration was observed. In some animals, IL-1 ra appeared to protect the architecture of cartilage and prevent sGAG loss while in other animals; IL-1 ra treatment did not prevent cartilage degeneration.

Synovial histopathology scores of H&E stained sections from the different experimental groups are presented in Fig. 4c. PBS-treated ACLT synovia exhibited a significantly higher degree of inflammation, hyperplasia and cellular infiltration compared to non-surgical controls (*p* < 0.001). PLGA IL-1 ra and IL-1 ra treatments following ACLT significantly reduced synovial pathology (*p* < 0.001) with no significant difference in the degree of synovial pathology between the two treatments. PLGA treatment alone had no disease modifying effect as the extent of synovial pathology in PLGA-treated animals was significantly higher than that in controls, PLGA IL-1 ra or IL-1 ra treated animals (*p* < 0.001), and not significantly different from PBS-treated animals. Representative H&E stained synovia from the different experimental groups is presented in Fig. 4d. PBS-treated ACLT synovium demonstrated synovial hyperplasia and thickening (as indicated by an arrow). This feature was also present in PLGA-treated ACLT synovium and to a lesser extent in IL-1 ra treated ACLT synovium. On the contrary, control and PLGA IL-1 ra treated ACLT synovium displayed normal appearing synovium.

**Fig. 5:**
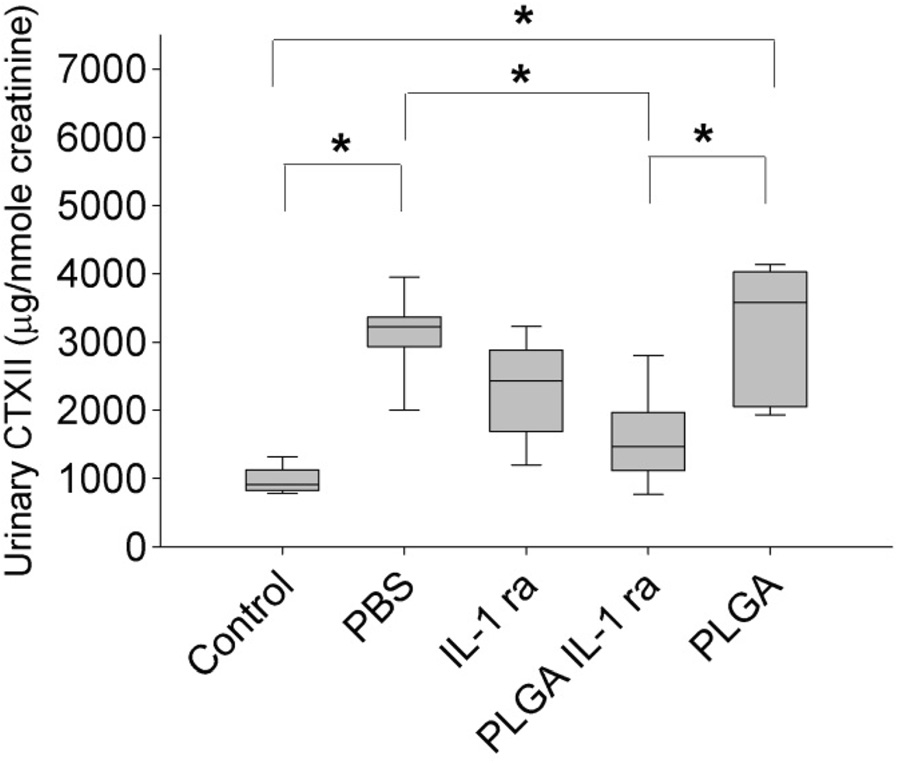
Urinary CTXII (uCTXII) concentrations, normalized to urinary creatinine level, collected over 24 h in control animals and animals undergoing ACLT and receiving weekly intra-articular injections of PBS (*n* = 7), IL-1 ra (*n* = 9), PLGA IL-1 ra (*n* = 9) or PLGA (*n* = 6) for 4 weeks starting one week following ACLT. PBS treated ACLT animals exhibited a significantly higher mean uCTXII concentration compared to control. Similarly, PLGA-treated ACLT animals exhibited a significantly higher mean uCTXII concentration compared to control. PLGA IL-1 ra treated ACLT animals demonstrated a significantly lower mean uCTXII concentration compared to PBS or PLGA-treated ACLT animals. *p < 0.001. Data represents average ± SD

The normalized uCTXII epitopes released in urine over a 24-h period from animals in the different experimental groups are presented in Fig. 5. PBS-treated ACLT animals demonstrated significantly higher uCTXII levels compared to controls (*p* < 0.001). IL-1 ra treatment did not significantly alter uCTXII levels compared to PBS treatment. On the contrary, PLGA IL-1 ra treatment significantly reduced uCTXII levels compared to PBS and PLGA treatments (*p* < 0.001). PLGA treatment alone had no disease modifying effect, as it did not significantly reduce uCTXII levels compared to PBS treatment.

## Discussion

In this study, we have successfully demonstrated the feasibility of encapsulating IL-1ra with superparamagnetic iron oxide in biodegradable PLGA microspheres. The incorporation of iron oxide allows the potential application of magnetic targeting to enhance PLGA IL-1 ra microsphere retention in arthritic joints (Butoescu et al. 2009a; Butoescu et al. 2009b). Using IL-1 ra as the encapsulated drug, a time-dependent release of IL-1 ra was observed with more than 90 % of IL-1 ra released over 3 days. An initial burst release of IL-1 ra, approximating one third of the total IL-1 ra protein was observed at 12 h following reconstitution. This initial burst is attributed to the porosity of the microparticles and the hydrophilic nature of the drug and not the degradation of the polymer (Lavi et al. 2007). Gorth et al. have shown that IL-1 ra can be successfully incorporated into PLGA microspheres. In their experiment, an estimated 60 % of released IL-1 ra occurred over the initial 3 days (Gorth et al. 2012). This difference in cumulative IL-1 ra release may be attributed to a difference in PLGA IL-1 ra particle size with our preparation having smaller particle sizes. One of the major challenges to encapsulating protein drugs is protein destabilization during preparation, storage and release processes (van de Weert et al. 2000). We have successfully encapsulated IL-1 ra with no loss to its bioactivity as demonstrated by the ability of the IL-1 ra released from the microspheres to inhibit lymphocyte proliferation and prevent sGAG release from cartilage explants to an extent similar to that of free IL-1 ra.

The encapsulation of IL-1 ra in a PLGA polymer slows the rate of IL-1 ra systemic clearance following intra-articular administration. Serum IL-1 ra concentrations were consistently higher, approximating a 10-fold difference, up to 6 h following free IL-1 ra treatment compared to PLGA encapsulated drug. This significant difference is explained by the fact that the majority of the drug remains encapsulated within the pharmaceutical polymer and thus is unavailable for systemic absorption. This is in contrast to the free IL-1 ra where virtually all of the drug is in the synovial fluid and is readily available for systemic absorption in a first-order kinetic process. Interestingly, at 24 h following IA administration, serum IL-1 ra concentrations in the PLGA IL-1 ra treated animals were significantly higher than in the free IL-1 ra treated animals. This difference can be due to the release of IL-1 ra from the PLGA microparticles and subsequent systemic absorption.

Using the rat ACLT model of PTOA, several of the key pathological changes to cartilage and synovium has been well documented (Caron et al. 1996; Elsaid et al. 2012; Jay et al. 2012; Elsaid et al. 2015). At 5 weeks following injury, cartilage surface roughness, reduced extracellular matrix proteoglycan staining and emerging loss of cartilage structural integrity, in the form of clefts, have been consistently observed in all PBS-treated animals. Overall, treatment with free IL-1 ra did not lead to a significant reduction in cartilage degeneration. While in some animals IL-1 ra did show a chondroprotective effect with preservation of cartilage matrix integrity, in the majority of animals, IL-1 ra treatment failed to provide any disease-modifying effect. In contrast, PLGA IL-1 ra treatment demonstrated a significant reduction in cartilage degeneration and preservation of extracellular matrix integrity. Across all IL-1 ra treatments, uCTXII levels were reduced compared to PBS-treated animals. uCTXII is a validated enzymatically-generated collagen type II degradation biomarker (Christgau et al. 2001). The reduction in uCTXII levels with IL-1 ra treatment provides supporting evidence that early blockade of IL-1 α effects following a traumatic joint injury can slow down the rate of cartilage degeneration.

Our understanding of the crosstalk between the synovium and cartilage in the pathogenesis of OA is evolving. Activation of toll-like receptors and the complement cascade in the synovial lining by cartilage degradation products plays a role in recruitment of inflammatory cells, release of cytokines and chemokines leading to cartilage erosion and osteophyte formation (Scanzello et al. 2008; Scanzello & Goldring 2012; de Lange-Brokaar et al. 2012). In the rat ACLT model, synovitis is evident at 5 weeks post-injury with significant inflammatory cell infiltration, increased vascularity and thickening of the synovial lining. PLGA IL-1 ra treatment has resulted in a significant reduction in synovial histopathology scores, due to a reduction in inflammatory cell infiltration and synovial thickening, compared to PBS treatment.

Taken together, our data suggests that encapsulating IL-1 ra in a PLGA polymer is feasible with no deleterious impact on its biological activity and that this encapsulation delays IL-1 ra systemic clearance following intra-articular administration. The delay in IL-1 ra systemic absorption may be responsible for the disease-modifying effect seen for PLGA IL-1 ra with prevention of cartilage degeneration and reduction in synovitis. Our future studies shall evaluate the impact of magnetic retention on enhancing the existing disease-modifying effect of intra-articular PLGA IL-1 ra, using a less frequent dosing strategy.

A limitation of this study is the use of an immortalized human lymphocyte cell line to evaluate the biological activity of PLGA encapsulated IL-1 ra. Additionally, we have not measured residual sGAG content in the bovine cartilage explants and the impact of IL-1 ra encapsulation on important and clinically relevant subchondral bone changes evident in PTOA was not evaluated. In the rat PTOA model, our IL-1 ra dosing strategy ameliorated synovitis but did not alter the extent of cartilage degeneration. This may be attributed to the delayed onset of treatment at one week following the traumatic insult compared with other reports that showed an effect of IL-1 ra with immediate onset of treatment following the insult (Caron et al. 1996; Elsaid et al. 2015; Furman et al. 2014).

## Conclusions

In summary, IL-1 ra is a promising pleiotropic disease-modifying anti-PTOA drug whose use is limited by brief joint residence time. We here in report on our success in encapsulating IL-1 ra in an inert biocompatible PLGA polymer that slowly degrades releasing the drug intra-articularly. This encapsulation has significantly improved the efficacy of the drug in a rat model of PTOA and provided supporting evidence to the utility of formulating IL-1 ra as a sustained release formulation for intra-articular delivery.

## Abbrevations

ACL: Anterior cruciate ligament
ACLT: Anterior cruciate ligament transection
IL-1 ra: Interleukin-1 receptor antagonist
IL-1: Interleukin-1
IL-6: Interleukin-6
N: Normal
NaOH: Sodium hydroxide
PLGA: Poly (lactide-co-glycolide)
PTOA: Posttraumatic osteoarthritis
SDS: Sodium dodecyl sulfate
TNFα: Tumor necrosis factor alpha
